# The Importance of Being Aware of Intrinsic Methods’ Limitation in Low-Density Lipoprotein Cholesterol Determination to Correctly Identify Cardiovascular Risk: Is Direct Determination Obtained with the Roche System Systematically Overestimating LDL in Very High-Risk Patients with Triglycerides Concentration of Less than 2.25 mmol/L?

**DOI:** 10.3390/jcm12134422

**Published:** 2023-06-30

**Authors:** Roberto Cemin, Simona Casablanca, Davide Ermacora, Massimo Daves

**Affiliations:** 1 Department of Cardiology, San Maurizio Regional Hospital of Bolzano (SABES-ASDAA), 39100 Bolzano, Italy; 2 Clinical Biochemical Laboratory, San Maurizio Regional Hospital of Bolzano (SABES-ASDAA), 39100 Bolzano, Italy

**Keywords:** cholesterol, LDL, cardiovascular risk

## Abstract

Background: low-density lipoprotein cholesterol (LDL-C) is a strong cardiovascular risk factor, but the methods used to correctly determine it are is still questioned. The aim of this study was to compare the direct determination of LDL-C levels, obtained with the Roche cobas c system, with LDL-C values calculated through Sampson’s and Friedewald’s equations in very high-risk patients with triglycerides concentrations of less than 2.25 mmol/L (<200 mg/dL). Methods: in 127 consecutive patients with a recent diagnosis of acute coronary syndrome and triglycerides of less than 2.25 mmol/L, plasma LDL-C was measured directly and calculated with Sampson’s and Friedewald’s equations before hospital discharge, and the data were compared. Results: median LDL values obtained with the Friedenwald and Sampson equations and with direct determination were 2.31 (IQR 1.59–3.21), 2.36 (IQR 1.66–3.26) and 2.64 (IQR 1.97–3.65) mmol/L, respectively. Direct measurements were higher by 0.35 and 0.40 mmol/L when compared to the levels calculated with Sampson’s or Friedewald’s equations, respectively (*p* < 0.01). The correlation between the two equations was almost perfect (rho 0.997) but decreased when the directly determined data were compared to those calculated with Sampson’s equation (rho 0.954) or Friedewald’s method (rho 0.939). Conclusion: direct determination generated higher values of LDL-C concentration through a probable systematic overestimation.

## 1. Introduction

Cardiovascular disease (CVD), especially atherosclerotic cardiovascular disease (ASCVD), is responsible for nearly 4 million deaths yearly in Europe, accounting for more than 60 million potential years of life lost to CVD annually [1]. It is also associated with relevant costs for diagnosis, treatment and rehabilitation.

Therefore, the latest European guidelines on Dyslipidaemias have once more stressed the importance of ASCVD prevention by promoting healthy lifestyle behaviour, with strong reductions in cardiovascular (CV) risk factors, such as low-density lipoprotein cholesterol (LDL-C) or blood pressure (BP), diabetes, and smoking status [2]. Numerous epidemiological studies, Mendelian randomization studies, and RCTs have consistently shown a log-linear relationship between the absolute LDL-C value or the total duration of exposure to LDL-C [3] and the risk of ASCVD [4,5], with significant CVD risk reductions due to LDL-C lowering. The recommended LDL-C concentration to be reached in patients with high cardiovascular risk who have already experienced an acute cardiovascular event is less than 1.42 mmol/L (55 mg/dL) or even lower (<1.14 mmol/L; <40 mg/dL) if a patient has had two cardiovascular events over a 24-month period [2].

With these rigorous recommendations, it is very important to standardize LDL-C determinations, also taking into consideration the fact that lipoprotein cholesterol concentrations differ significantly when measured in fed or in fasted subjects [6] and that the latest evidence supports the use of non-fasting lipid testing for most of the subjects [7]. 

It is also mandatory to entrust a reliable laboratory method for lipid and lipoproteins estimation to guide therapeutic decisions, especially in patients on the recent most effective lipid-lowering therapy, such as proprotein convertase subtilisin/kexin type 9 (PCSK9) inhibitors, which can markedly decrease LDL-C concentration [8].

The β-quantification is considered the gold standard for LDL-C measurement, but it is time consuming and expensive and almost never used in daily clinical practice. Surrogate methods are indirect determinations obtained through formulas that extrapolate the LDL-C from other lipid parameters. The well-known Friedewald formula [9] is used all around the world. Its main source of error is the Triglycerides/5 ratio, which represents an estimate of very-low-density lipoprotein cholesterol (VLDL-C) and can vary considerably, diminishing the accuracy of the formula for high-triglyceride (TG) samples (>4.52 mmol/L; >400 mg/dL).

Martin et al. [10,11] developed an equation based on the Vertical Auto Profile test, which is a vertical-rotor ultracentrifugation-based method [12]. They replaced the fixed TG denominator of 5 used in the Friedewald equation with an empirical denominator that varies depending on concentrations of TGs and non-high-density lipoprotein cholesterol (HDL-C) [10], reporting greater accuracy than the Friedewald formula, particularly for low-LDL-C samples or high TG levels. The Vertical Auto Profile method, on which the Martin formula is based, may sometimes underestimate VLDL-C levels in high-TG samples [12,13], and this equation has never been validated for patients with hypertriglyceridemia [10,11]. For all of these reasons and because of the complexity of the method, the Martin equation is not routinely used.

Recently, Sampson et al. [14] developed a new equation, which proved to be more accurate than Friedewald and Martin equations when compared with β-quantification, particularly for patients with hypertriglyceridemia. It calculates the LDL-C concentration in patients with TG concentrations of up to 9 mmol/L (800 mg/dL) as accurately as the Friedewald equation does for TG concentrations of less than 4.5 mmol/L (400 mg/dL). As an alternative to calculating LDL-C, non-HDL-C can also be derived by subtracting HDL-C from the total cholesterol (TC), obtaining a measure of the TC carried by all atherogenic ApoB-containing lipoproteins, including TG-rich particles in VLDL and their remnants [15], LDL-C and the two minor lipoprotein classes: intermediate-low density lipoprotein (IDL) and Lipoprotein a (Lp(a)).

To overcome the problems associated with the calculation of LDL-C, in recent years, much effort has been put into the development of tests for the direct measurement of LDL-C (dLDL-C) [16,17], which should be a much easier method to obtain the result. Despite this, it is still common practice to calculate LDL-C because of the extra cost of adding dLDL-C to the lipid panel and the poor analytical performance of some dLDL-C tests [18,19]. Furthermore, it would be very important to know the reliability of the method used to determine LDL-C concentrations to correctly identify actual cardiovascular risk and, subsequently, guide therapy.

The aim of our study was to compare direct LDL-C concentration determination to data calculated using the new Sampson’s equation and Friedewald formula in very-high-risk patients with TG levels of less than 2.25 mmol/L (200 mg/dL) in order to highlight possible differences that should be known and taken in account in clinical practice. 

## 2. Material and Methods

In this observational study, LDL-C concentrations were determined and calculated in 130 consecutive patients discharged with the diagnosis of acute coronary syndrome from the Department of Cardiology of the San Maurizio Regional Hospital of Bolzano, Italy, during the 4-month period between 1 February and 31 May 2018. All the patients had undergone a percutaneous coronary intervention with complete revascularisation during their hospital stay. They were then followed up in our outpatient coronary clinic during the subsequent six months. Having already experienced an acute coronary syndrome, all these patients were considered to have very high cardiovascular risk and put on a combination of high-efficacy statins, ezetimibe or PCSK9 inhibitors. 

Written informed consent for the evaluation of the clinical data accrued during the admission was obtained from all patients, and the study was conducted according to the latest version of the Declaration of Helsinki given by the World Medical Association. Formal approval of the protocol by the local Ethics Committee was considered unnecessary because the study did not interfere with the usual clinical routine, as the assessment of lipid status was part of the routine examinations prescribed to patients after the acute event, and the data were handed anonymously. 

In all patients, plasma LDL-C was measured before hospital discharge with LDL-Cholesterol Gen.3 (Roche Diagnostics GmbH, Mannheim, Germany), an in vitro test for the quantitative determination of LDL-cholesterol in human serum and plasma on Roche/Hitachi cobas c systems (Roche Diagnostics). LDL-Cholesterol Gen.3 is a homogeneous enzymatic colorimetric assay in which cholesterol esters and free cholesterol in LDL are measured on the basis of a cholesterol enzymatic method using cholesterol esterase and cholesterol oxidase in the presence of surfactants that selectively solubilize only LDL; surfactants and a sugar compound contained in the assay inhibit the enzyme reactions to the lipoproteins other than LDL, and, for this reason, the cholesterol contained in HDL, VLDL and chylomicron is not determined (LDL-Cholesterol Gen.3 2017-06, V 3.0-English. Roche Diagnostics GmbH, Mannheim, Germany). All measurements were performed on the Roche cobas c 502 analyser following the manufacturer’s instructions.

Aside from this direct determination, LDL-C was also calculated using the Sampson equation and Friedewald formula (reported below), and the results obtained with the three methods were compared to each other in 127 of the 130 patients. Three patients were excluded from the analysis because they had triglycerides concentration of more than 2.25 mmol/L (200 mg/dL), therefore, theoretically leading to some inaccuracy in the Friedewald formula. The choice of excluding patients with higher triglyceride values was made in order to avoid even minimal interference biases in the Friedewald formula.

*Friedewald formula: LDL-C =Total Cholesterol-HDL-TG/5*

*This applies when the concentrations of the associated lipid parameters are expressed in mg/dL, while if the concentrations are expressed in mmol/L, a factor of 2.2 (TG/2.2 instead of TG/5) must be used.*

*Sampson Equation:*

*LDL-C = Total Chol/0.948 − HDL/0.971 − (TG/8.56 + TG × nonHDL/2140 − TG^2^/16100) − 9.44*

## 3. Statistical Analyses 

Continuous variables were described using the range, means and standard deviations if normally distributed and medians and interquartile ranges if not. The departure from normality of the LDL-C levels was assessed using the skewness and kurtosis tests for normality (Shapiro–Wilk Test for normal distribution). Data on LDL-C were inspected using scatter plots and adding both linear and non-linear fitting functions. The agreement between different LDL-C values obtained in the same patients with the two different equations and direct determination was assessed using Lin’s concordance correlation coefficient for agreement rho on a continuous measure [20,21], which is the product of Pearson correlation coefficient and a bias factor measuring accuracy using the Stata 14 (StataCorp, College Station, TX, USA). The Wilcoxon test was used to assess the differences between the methods, and Passing-Bablok regression was carried out to analyse the numerical results obtained with the two equations and direct measure. A *p*-value of less than 0.05 was considered statistically significant. Bland and Altman plots [22] were generated, displaying the limits of agreement of the different methods. Wilcoxon’s test, Passing-Bablok regression and the Bland–Altman plots were performed using MedCalc v. 17.4.4 statistical software (MedCalc Software, Ostend, Belgium). 

## 4. Results

The clinical and demographic characteristics of the patients enrolled are reported in Table 1. Seventy-two were men, and fifty-five were females, with a median age of 69 years (min, 33; max, 94 years). 

The median LDL-C values obtained with the Friedenwald and Sampson equations and with direct determination were 2.31 (IQR 1.59–3.21), 2.36 (IQR 1.66–3.26) and 2.64 (IQR 1.97–3.65) mmol/L (89.4 (IQR 61.4–124.0), 91.2 (IQR 64.2–126.1) and 102.0 (IQR 76.2–141.0) mg/dL), respectively. Direct LDL-C measurements were higher by 0.35 (IQR 0.25–0.45) and 0.40 (IQR 0.29–0.49) mmol/L (13.4 (IQR 9.7–17.4) and 15.6 (IQR 11.2–19.2) mg/dL) when compared to the levels calculated with Sampson’s or Friedewald’s equations, respectively (*p* < 0.001 for all comparison).

Being these patients at very high cardiovascular risk, the target LDL-C to be reached would be less than 1.4 mmol/L (55 mg/dL), with a 50% reduction in baseline values. The resulting LDL-C values were already inferior to 1.4 mmol/L in 18 and 20 patients using Sampson’s and Friedewald’s equations, respectively, and only in 12 when LDL-C was measured directly with the Roche system.

**Figure 1:**
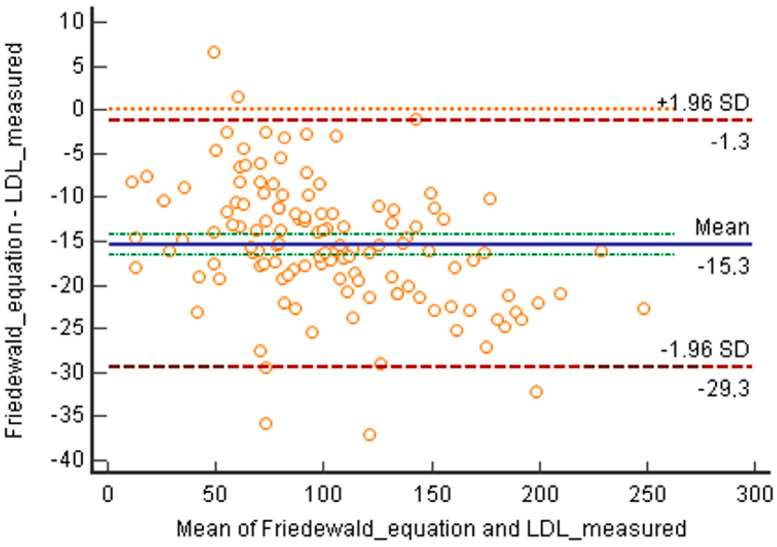
Bland–Altmann plot comparing LDL values directly measured with those calculated using Friedewald’s equation. Mean difference: −0.39 mmol/L (−15.3 mg/dL).

The Bland–Altmann plot comparing LDL-C directly measured with the results calculated using Friedewald’s equation showed a negative bias, indicating higher values for the direct determination and a mean difference of −0.39 mmol/L (−15.3 mg/dL) (Figure 1). 

**Figure 2:**
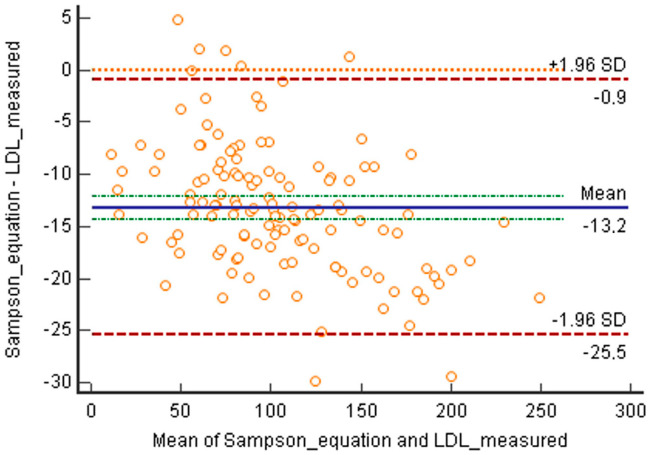
Bland–Altmann plot comparing LDL values directly measured with those calculated with Sampson’s equation. Mean difference: −0.34 mmol/L (−13.2 mg/dL).

A similar negative bias was also found with Sampson’s equation, showing higher values for direct determination and a mean difference of −0.34 mmol/L (−13.2 mg/dL) (Figure 2), whilst the differences between the data obtained through the two equations were minimal (mean difference −0.05 mmol/L (−2.1 mg/dL)), with slightly higher values obtained with Friedewald’s equation.

Direct LDL-C measurement (variable y) was linearly related (Cusum test n.s.) to the data obtained with Friedewald’s formula (variable x) according to Passing-Bablok regression as y = 9.048 (95% CI 7.019 to 11.330) + 1.0656 (95% CI 1.043 to 1.092) X. A similar relation was found between direct LDL-C measurement and the data obtained with Sampson’s equation (variable z): y = 7.924 (95% CI 5.759 to 10.209) + 1.059 (95% CI 1.037 to 1.081) Z. The Passing-Bablok regressions indicate constant and proportional differences between the measured and calculated value of LDL-C with both equations, whilst the data derived from both equations showed good agreement. The correlation between the data obtained with the two equations (Friedewald and Sampson) was almost perfect (rho 0.997). 

**Figure 3:**
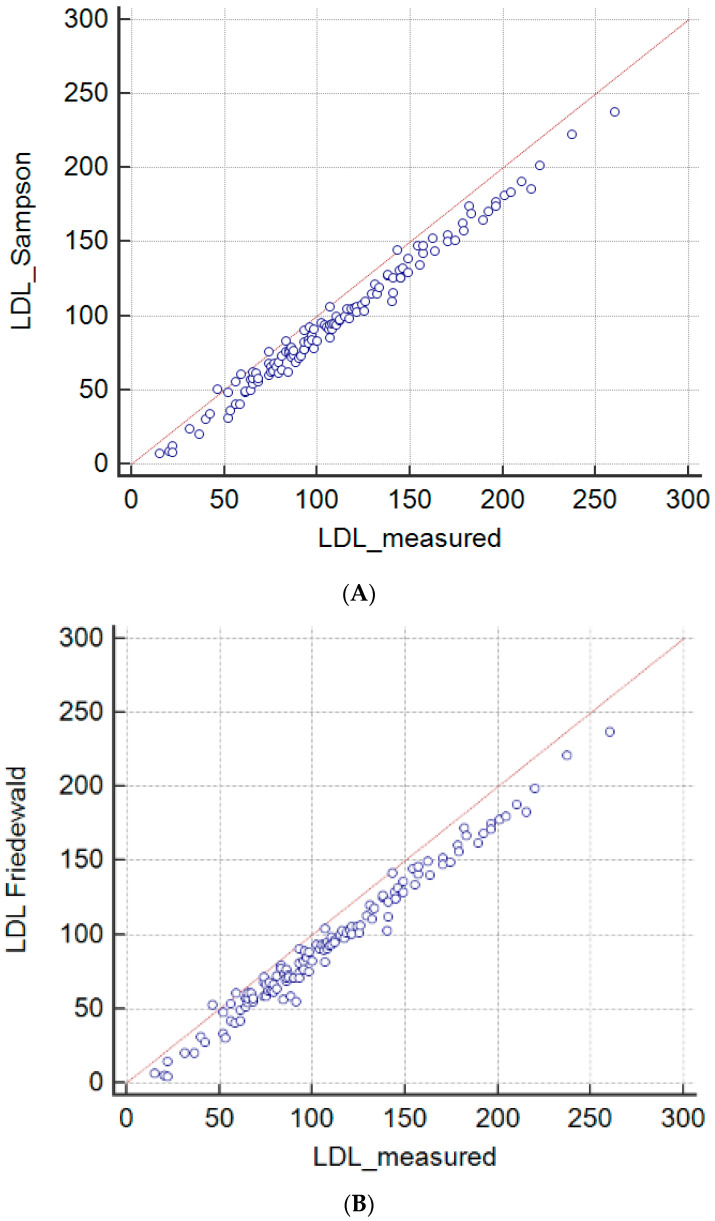
Lin’s concordance correlation of LDL values obtained with Sampson’s equation and directly measured: rho 0.954 (substantial) (**A**) and correlation of LDL values obtained with Friedewald’s equation and directly measured: rho 0.939 (moderate) (**B**).

It decreased to substantial (rho 0.954) when the directly determined data were compared to those calculated using Sampson’s equation and only to moderate (rho 0.939) when the direct determination was compared to data obtained with Friedewald’s method (Figure 3A,B). 

## 5. Discussion

Our results confirm previous observations of less reliability in terms of direct LDL-C determination when compared to Friedewald or Sampson equations in very-high-risk patients with TG levels of less than 2.2 mmol/L (<200 mg/dL), in whom both formulae have been considered very accurate [23]. Direct determination obtained with the Roche method leads to a probable overestimation of LDL-C with respect to the values obtained with the two formulas, at least in the high-risk population covered by our study, which could have implied further pharmacological efforts to reduce these levels. 

Very important clinical decisions are routinely taken concerning the LDL-C values provided by clinical laboratories, which are often estimated with the Friedewald formula. This calculation is not reliable if the patient is not fasting or if the serum TG values are >4.5 mmol/L (>400 mg/dL) [9], but it becomes increasingly inaccurate with TG concentrations of more than 2.2 mmol/L (200 mg/dL) [23]. Higher levels of TGs (>2.2 mmol/L; >200 mg/dL) lead to an underestimate of LDL-C when calculated using the Friedewald formula, particularly at LDL-C values of less than 70 mg/dL and to an overestimation if the LDL-C is more than 130 mg/dL [24]. 

The LDL-C reference method would require the ultracentrifugation of the plasma for 18 h at its own density (1.006 g/mL), the measurement of cholesterol in the 1.006 g/mL infranatant fraction and the subtraction of HDL-C from this value to obtain the LDL-C concentration [17]. This method is not practicable in the routine of modern clinical laboratories, with large workloads and the need for rapid reports. Therefore, direct homogeneous LDL-C assays without sample pre-treatment that can be performed on the clinical chemistry analysers in complete automation were developed, but their reliability is still questioned, as confirmed by our observations. 

In some studies, calculated LDL-C and direct LDL-C showed very strong correlations, but calculated LDL-C can underestimate LDL-C for TG >177 mg/dL (2 mmol/L) and may be misleading at very low LDL-C levels, especially in the presence of high TG [24,25,26,27,28]. Another study, performed on 27,331 women, showed that direct LDL-C measurements were lower by 5–10 mg/dL compared with Friedewald fasting measurements [16].

The latest American guidelines on the management of blood cholesterol recommend the measurement of direct LDL-C or modified LDL-C estimates with the Martin equation in class IIa to improve accuracy over the Friedewald formula in patients with LDL-C levels less than <1.8 mmol/L (<70 mg/dL) [29,30]. No recommendation on the manufacturers’ methods to be used is mentioned, and this seems unwise when considering our and previous studies. Having lower or higher LDL-C values measured by direct methods may consequently lead to the misclassification of some individuals and represents an important issue to be considered for the correct stratification of ASCVD risk. 

Differently, direct HDL-C assays are universally performed by routine clinical laboratories because the test is fully automated and thus avoids the labor-intensive manual precipitation step used to remove LDL before measuring HDL-C [31]. Measuring non-HDL-C could overcome the problems generated in LDL-C calculations.

The new Sampson equation should be more reliable when compared to the Friedewald formula, especially in the case of high TG levels [14], but our data do not confirm this observation in terms of patients with very high CV risk and low LDL-C and TG levels, where both equations showed excellent correlation. 

Different from the American guidelines, the latest European guidelines on dyslipidaemias state that plasma LDL-C can be either calculated or measured directly [2]. Several studies have shown that in some circumstances (people with elevated TG concentrations, diabetes, obesity, or very low LDL-C concentrations), both calculated and directly measured LDL-C may underestimate the total concentration of cholesterol carried by LDL-C and also underestimate the total concentration of ApoB-containing lipoproteins, thus underestimating the risk of ASCVD [28,32]. Considering the potential inaccuracy of LDL-C values among these categories of patients, the European guidelines recommended the measurement of both ApoB and non-HDL-C as part of routine lipid analysis for risk evaluation in patients with elevated plasma TGs [2]. Moreover, considering the accuracy that ApoB provides for the estimation of the total concentration of atherogenic particles under all circumstances, the European Guidelines defined ApoB as the preferred measurement to further refine the estimate of ASCVD risk that is modifiable using lipid-lowering therapy [2]. 

Other indexes, such the triglyceride–glucose index (TyG), have been recently proposed for a more exhaustive risk classification in specific populations, such as patients with diabetes and acute coronary syndrome [33]. The TyG index seems to be a useful marker for predicting future events in patients with diabetes and acute coronary syndrome independently of other known cardiovascular risk factors.

In our study, the direct determination with the Roche system generated higher values of LDL-C when compared to the data obtained through the Friedewald equations in patients with low TG, where it has traditionally proven excellent accuracy. The Sampson equation confirmed the reliability of the Friedewald formula, leading us to hypothesize a systematic overestimation of direct determination, even if it should be kept in mind that formulas are not the gold standard but only the best approximation known. 

This discrepancy should also be taken into consideration in registries on lipid control, as in the recently published SANTORINI [34], where speculations are made on the percentages of patients reaching LDL-C targets, but no mention is made of the method used to obtain the LDL-C value. Only the study presenting paper [35] stated that there was no specified standardized lipid measurement, introducing inherent variances into laboratory tests, which will lead to a degree of variability into the results. Specifying a clear LDL-C determination method would generate more reliable results, which could otherwise simply depend on different methods used. 

The optimal management of patients requiring lipid-lowering therapy should be based on reliable laboratory methods, particularly in high- or very-high-risk patients. It is, therefore, essential to rely on a validated method as much as possible, reflecting the gold standard process. As recently reported by Choi et al., in order to accurately evaluate LDL-C, a laboratory-specific equation together with direct determination is needed [36]. Physicians should be aware of the possible higher results of direct determination of LDL-C when interpreting the results and making medical decisions. 

It is always necessary to know the limits of the method used in our laboratory for LDL-C determination because a wrong evaluation of the lipid profile can mislead physicians. In our case, being aware of the possible systematic overestimation of the Roche system could suggest preferring indirect calculation through Friedenwald or Sampson formulas.

The main limit of our observations is the small number of the patients included, which is partially counterbalanced by the very strict selection of the population.

## 6. Conclusions

Our study shows that direct determination generated higher values of LDL-C concentration through a probable systematically overestimation when compared to values obtained through Sampson’s and Friedewald’s equations, which showed an excellent reliability in patients within very-high-risk patients and TG levels of less than 2.2 mmol/L (<200 mg/dL). 

It is always necessary to be well aware of the limits of the method used in our laboratory for LDL-C determination in order to avoid misclassification errors, which could lead to inappropriate therapy. Even international studies or registries on lipid-lowering therapy should take into consideration the possible discrepancies deriving from different methods used in LDL-C evaluation.

## Figures and Tables

**Table 1:** Clinical and demographic characteristics of the patients enrolled.

Patients	127
Sex (male/female)	72 (57%)/55 (43%)
Age years (min-Max)	69 (33–94)
Very high risk	100%
PCSK9i	28 (22%)
LDL-C median (95% CI) (IQR) mmol/L Friedewald	2.31 (2.13 to 2.65) (1.6–3.2)
LDL-C median (95% CI) (IQR) mmol/L Sampson	2.36 (2.16 to 2.71) (1.66–3.26)
LDL-C median (95% CI) (IQR) mmol/L Roche	2.64 (2.48 to 3.07) (1.97–3.65)

PCSK9i: proprotein convertase subtilisin/kexin type 9 inhibitors. CI: Confidence Interval.

## Data Availability

Original anonymized data could be supplied upon request.
